# Supplementary material for: Using the antibody-antigen binding interface to train image-based deep neural networks for antibody-epitope classification
Source: PLoS Comput Biol. 2021 Mar 29;17(3):e1008864. doi: 10.1371/journal.pcbi.1008864 (PMC8032195; doi:10.1371/journal.pcbi.1008864)
Supplement: S1 Text — (DOCX) [file pcbi.1008864.s001.docx]

**S1 Text: *Description of the set of anti-EBOV Abs***

To assess the magnitude of the human antibody response to EBOV glycoprotein (GP), Bornholdt *et al*. (2016) cloned an extensive panel of mAbs targeting GP from peripheral B cells of a convalescent donor that survived the 2014 EBOV outbreak in Zaire [1]. The researchers found that approximately 3% of IgG+ B cells were specific for the EBOV GP ectodomain. Further analysis of the heavy and light-chain variable regions of the 349 cloned mAbs targeting the GP ectodomain revealed a highly diverse repertoire containing 294 independent clonal lineages. Many mAbs from the set exhibited high potency and 77% of them could neutralize live EBOV. Sequences of heavy- and light-chains of these 349 mAbs can be found in GenBank under accession numbers listed in Table S7 of reference [1].

Furthermore, Bornholdt *et al*. (2016) performed competitive binding assays together with single-particle electron microscopy (EM) to investigate the epitopes in EBOV GP targeted by these mAbs and characterize their complexes. These experiments showed that the anti-GP antibody response in this patient was primarily directed to four non-overlapping sites on fully glycosylated EBOV GP ectodomain, as the majority of the mAbs could be assigned to four major competition groups. These mAbs included ADI-15731 (Fig 6D), a 13C6 competitor that binds to the glycan cap. Two KZ52 competitors: ADI-15762 that binds within the glycan cap, and ADI-15734 (Fig 6B) that binds to the central region of the GP trimer. ADI-15758 (Fig 6C), an ADI-15974 competitor that also bind to a region proximal to the viral membrane, and ADI-15859, an ADI-15810 competitor that binds within the GP stalk region. Subsequent studies [2-10] have provided a finer map of the main Ab binding regions in EBOV GP, and showed that neutralizing mAbs preferentially target the GP1 head, fusion loop, base, and stalk (HR2) regions presented after enzymatic cleavage of GP.

**References**

1. Bornholdt ZA, Turner HL, Murin CD, Li W, Sok D, Souders CA, et al. Isolation of potent neutralizing antibodies from a survivor of the 2014 Ebola virus outbreak. Science. 2016;351(6277):1078-83.

2. Wec AZ, Bornholdt ZA, He S, Herbert AS, Goodwin E, Wirchnianski AS, et al. Development of a human antibody cocktail that deploys multiple functions to confer pan-ebolavirus protection. Cell Host & Microbe. 2019;25(1):39-48.e5.

3. Wec AZ, Herbert AS, Murin CD, Nyakatura EK, Abelson DM, Fels JM, et al. Antibodies from a human survivor define sites of vulnerability for broad protection against ebolaviruses. Cell. 2017;169(5):878-90.e15.

4. West BR, Wec AZ, Moyer CL, Fusco ML, Ilinykh PA, Huang K, et al. Structural basis of broad ebolavirus neutralization by a human survivor antibody. Nat Struct Mol Biol. 2019;26(3):204-12.

5. Saphire EO, Schendel SL, Fusco ML, Gangavarapu K, Gunn BM, Wec AZ, et al. Systematic analysis of monoclonal antibodies against Ebola virus GP defines features that contribute to protection. Cell. 2018;174(4):938-52.e13.

6. Davis CW, Jackson KJL, McElroy AK, Halfmann P, Huang J, Chennareddy C, et al. Longitudinal analysis of the human B cell response to Ebola virus infection. Cell. 2019;177(6):1566-82.e17.

7. Ehrhardt SA, Zehner M, Krähling V, Cohen-Dvashi H, Kreer C, Elad N, et al. Polyclonal and convergent antibody response to Ebola virus vaccine rVSV-ZEBOV. Nat Med. 2019;25(10):1589-600.

8. Ilinykh PA, Santos RI, Gunn BM, Kuzmina NA, Shen X, Huang K, et al. Asymmetric antiviral effects of ebolavirus antibodies targeting glycoprotein stem and glycan cap. PLoS Pathog. 2018;14(8):e1007204.

9. Zhang Q, Gui M, Niu X, He S, Wang R, Feng Y, et al. Potent neutralizing monoclonal antibodies against Ebola virus infection. Sci Rep. 2016;6:25856.

10. Corti D, Misasi J, Mulangu S, Stanley DA, Kanekiyo M, Wollen S, et al. Protective monotherapy against lethal Ebola virus infection by a potently neutralizing antibody. Science. 2016;351(6279):1339-42.
