# Supplementary material for: Using the antibody-antigen binding interface to train image-based deep neural networks for antibody-epitope classification
Source: PLoS Comput Biol. 2021 Mar 29;17(3):e1008864. doi: 10.1371/journal.pcbi.1008864 (PMC8032195; doi:10.1371/journal.pcbi.1008864)
Supplement: S1 Fig — (A) Traditional image recognition problem in which a DNN model is used to identify or classify objects, e.g., cats from dogs, different breeds, etc. Images were obtained from Wikimedia Commons (see S9 Table for a list of credits and reproduction license agreements). (B) For the goal of developing AI techniques capable of Ab characterization based on B cell sequence, we need to develop a number of related methodological capabilities that included conversion of sequences into image representations to enable identification and classification by means of DNNs. (DOCX) [file pcbi.1008864.s002.docx]

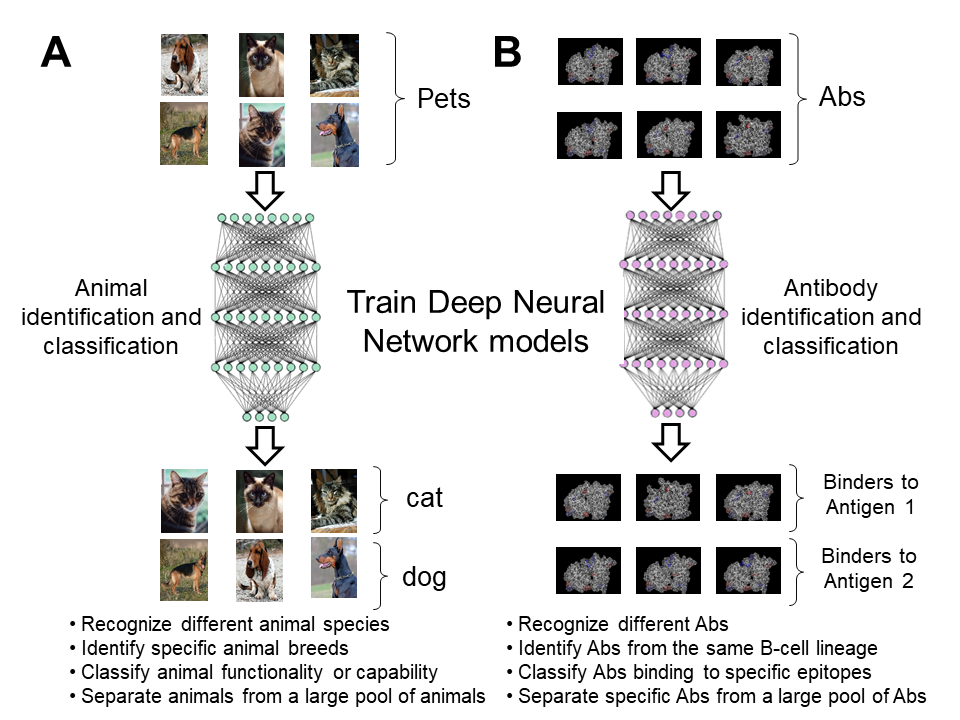


**S1 Fig. *Proposed approach to assess high-throughput B cell immune-sequencing data with Artificial Intelligence methods.***

(A) Traditional image recognition problem in which a DNN model is used to identify or classify objects, e.g., cats from dogs, different breeds, etc. Images were obtained from Wikimedia Commons (see S9 Table for a list of credits and reproduction license agreements). (B) For the goal of developing AI techniques capable of Ab characterization based on B cell sequence, we need to develop a number of related methodological capabilities that included conversion of sequences into image representations to enable identification and classification by means of DNNs.
