# Supplementary material for: Using the antibody-antigen binding interface to train image-based deep neural networks for antibody-epitope classification
Source: PLoS Comput Biol. 2021 Mar 29;17(3):e1008864. doi: 10.1371/journal.pcbi.1008864 (PMC8032195; doi:10.1371/journal.pcbi.1008864)
Supplement: S2 Fig — Family lineages are labeled L1 to L10. Fingerprints corresponding to all Ab members of a family are displayed in a column. The order in which Abs are listed in a family is arbitrary. Note: the Abs labels have been simplified where “A” stands for “ADI-”. The ID numbers correspond to the Abs described in reference [23]. (DOCX) [file pcbi.1008864.s003.docx]

**
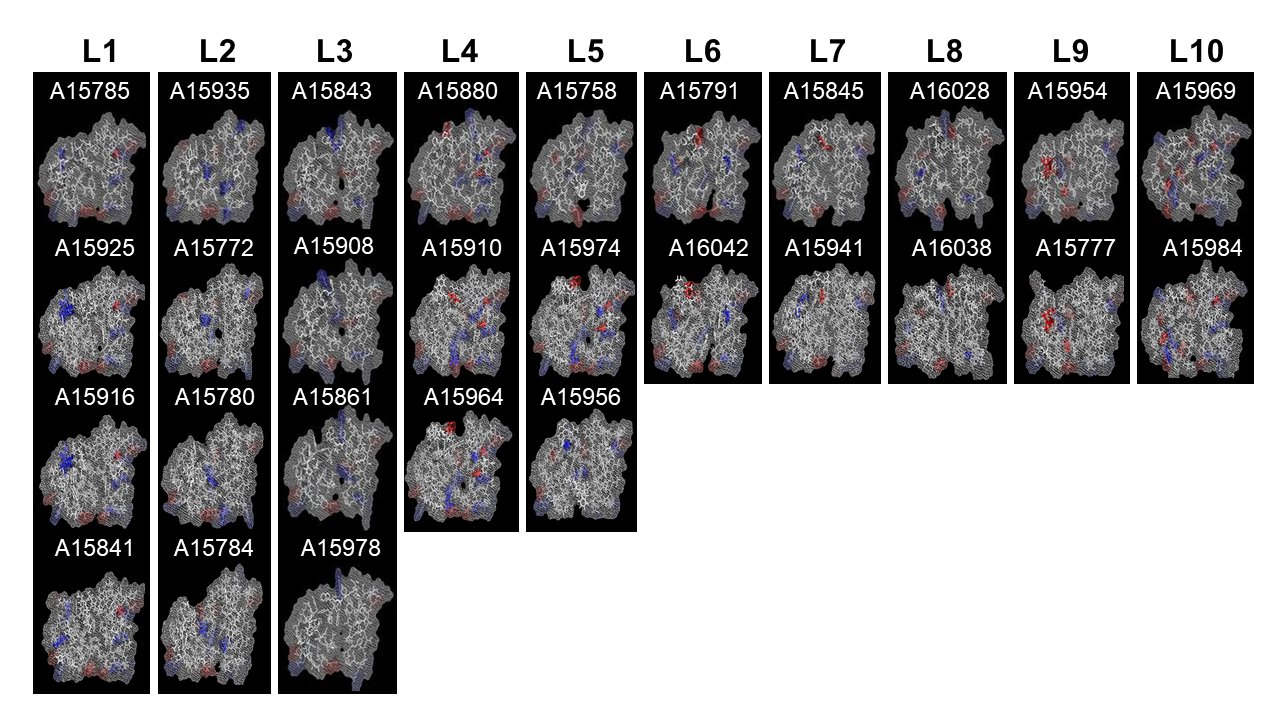
**

**S2 Fig: *Fingerprints of twenty-eight anti EBOV Abs associated with ten family lineages.***

Family lineages are labeled L1 to L10. Fingerprints corresponding to all Ab members of a family are displayed in a column. The order in which Abs are listed in a family is arbitrary. *Note*: the Abs labels have been simplified where “A” stands for “ADI-”. The ID numbers correspond to the Abs described in reference [1].

**References**

1. Bornholdt ZA, Turner HL, Murin CD, Li W, Sok D, Souders CA, et al. Isolation of potent neutralizing antibodies from a survivor of the 2014 Ebola virus outbreak. Science. 2016;351(6277):1078-83.
