# Supplementary material for: Using the antibody-antigen binding interface to train image-based deep neural networks for antibody-epitope classification
Source: PLoS Comput Biol. 2021 Mar 29;17(3):e1008864. doi: 10.1371/journal.pcbi.1008864 (PMC8032195; doi:10.1371/journal.pcbi.1008864)
Supplement: S1 Table — The ID numbers correspond to the Abs described in reference [23]. (DOCX) [file pcbi.1008864.s004.docx]

S1 Table. *Identification numbers of anti EBOV antibodies from ten family from the most populated lineages.* The ID numbers correspond to the Abs described in reference [1].

| Ab family lineage | Member 1 | Member 2 | Member 3 | Member 4 |
| --- | --- | --- | --- | --- |
| 1 | 15785 | 15925 | 15916 | 15841 |
| 2 | 15935 | 15772 | 15780 | 15784 |
| 3 | 15843 | 15908 | 15861 | 15978 |
| 4 | 15880 | 15910 | 15964 |  |
| 5 | 15758 | 15974 | 15956 |  |
| 6 | 15791 | 16042 |  |  |
| 7 | 15845 | 15941 |  |  |
| 8 | 16028 | 16038 |  |  |
| 9 | 15954 | 15777 |  |  |
| 10 | 15966 | 15951 |  |  |

**References**

1. Bornholdt ZA, Turner HL, Murin CD, Li W, Sok D, Souders CA, et al. Isolation of potent neutralizing antibodies from a survivor of the 2014 Ebola virus outbreak. Science. 2016;351(6277):1078-83.
