# Supplementary material for: Using the antibody-antigen binding interface to train image-based deep neural networks for antibody-epitope classification
Source: PLoS Comput Biol. 2021 Mar 29;17(3):e1008864. doi: 10.1371/journal.pcbi.1008864 (PMC8032195; doi:10.1371/journal.pcbi.1008864)
Supplement: S2 Table — Sequence information summary from BRILIA [24] for 28 anti-EBOV antibodies used to train and test DNN models for family lineage detection. The charged residues found within the CDRs are highlighted in the respective columns listing the sequences. (DOCX) [file pcbi.1008864.s005.docx]

S2 Table. *Sequence analysis of anti EBOV antibodies from ten family lineages*.

Sequence information summary from BRILIA [1] for 28 anti-EBOV antibodies used to train and test DNN models for family lineage detection. The charged residues found within the CDRs are highlighted in the respective columns listing the sequences.

| Lineage | Ab Id | CDRH1 | CDRH2 | CDRH3 | VH Germline | DH Germline | JH Germline | CDRL1 | CDRL2 | CDRL3 | VL Germline | JL Germline | |
| --- | --- | --- | --- | --- | --- | --- | --- | --- | --- | --- | --- | --- | --- |
| 1 | 15916 | GFTFNSYG | ISYDGSNK | AKGAVVIAMLNYFDY | IGHV3-30*18\| IGHV3-30-5*01 | IGHD2-21*01 | IGHJ4*02 | QSVLYSSKNKNY | RAS | QQYYSTPQT | IGKV4-1*01 | IGKJ1*01 |  |
| 1 | 15925 | GFTFSSYG | ISYDGSNK | AKGAVVIAMLNYFDS | IGHV3-30*18\| IGHV3-30-5*01 | IGHD2-21*01 | IGHJ4*02 | QSVLYSSKNKNY | RAS | QQYYSTPQT | IGKV4-1*01 | IGKJ1*01 |  |
| 1 | 15785 | GFTFSNYG | ISYDGSIK | AKGTVIIAMLNYFDY | IGHV3-30*18\| IGHV3-30-5*01 | IGHD2-21*01 | IGHJ4*02 | QGVLYSSNNKNY | GTS | QQYYSTPQT | IGKV4-1*01 | IGKJ1*01 |  |
| 1 | 15841 | GFTFSSYA | ISYDGSIK | AKGAVVIAMLNYFDY | IGHV3-30*18\| IGHV3-30-5*01 | IGHD2-21*01 | IGHJ4*02 | CKSSQHSNNKNY | RAS | QQYYNTPQT | IGKV4-1*01 | IGKJ1*01 |  |
| 2 | 15780 | GYTFTSYG | ISAYNGNT | ARDLTPRYGMDV | IGHV1-18*04 | IGHD3-9*01 | IGHJ6*02 | QSLLHSNGYNY | MGS | MQALQTPRT | IGKV2-28*01\| IGKV2D-28*01 | IGKJ2*02 |  |
| 2 | 15784 | GYTFSSYG | ISAYNGYT | ARDLTPRDGMDV | IGHV1-18*04 | IGHD3-9*01 | IGHJ6*02 | QSLLHSNGYNY | MGS | MQALQTPRT | IGKV2-28*01\| IGKV2D-28*01 | IGKJ2*02 |  |
| 2 | 15935 | GYTFRNYG | ISAYNGYT | ARDLTPRYGLDV | IGHV1-18*04 | IGHD3-9*01 | IGHJ6*02 | QSLLHSNGYNY | MGS | MQALQSPWT | IGKV2-28*01\| IGKV2D-28*01 | IGKJ2*02 |  |
| 2 | 15772 | GYTFTTYG | ISAYNGNT | ARDLTPRYGMDV | IGHV1-18*04 | IGHD3-9*01 | IGHJ6*02 | QSLLHSNGYNY | MGS | MQALQTPRT | IGKV2-28*01\| IGKV2D-28*01 | IGKJ2*02 |  |
| 3 | 15843 | GGSFSGYS | INHSGST | ARAWLRSRGYPSFDY | IGHV4-34*01 | IGHD6-13*01 | IGHJ4*02 | QSLLHSNGYNY | MGS | MQALQTLT | IGKV2-28*01\| IGKV2D-28*01 | IGKJ5*01 |  |
| 3 | 15908 | GGSFSGYY | INHSGST | ARRLQRHGNYVGSFDY | IGHV4-34*01 | rIGHD2/ OR15-2a*01\| rIGHD2/ OR15-2b*01 | IGHJ4*02 | QSLLHNNGYNY | MGS | MQALQAPVT | IGKV2-28*01\| IGKV2D-28*01 | IGKJ5*01 |  |
| 3 | 15978 | GGSFSGYY | INHSGST | ARAWLRTSWYPSFDY | IGHV4-34*01 | IGHD6-13*01 | IGHJ4*02 | QSLLHSNGYNY | MGS | MQALQTLT | IGKV2-28*01\| IGKV2D-28*01 | IGKJ5*01 |  |
| 3 | 15861 | GGSFSGYH | INHSGST | ARAWLRSSSYPSFDY | IGHV4-34*01 | IGHD6-13*01 | IGHJ4*02 | QSLLHSNGYNY | MGS | MQALQTLT | IGKV2-28*01\| IGKV2D-28*01 | IGKJ5*01 |  |
| 4 | 15880 | GFTFSRYW | IKQDGSEK | AREAFLEWLAPLGHYYMDV | IGHV3-7*03 | IGHD3-3*02 | IGHJ6*03 | QSVSSSY | GAS | QLYGSSFRT | IGKV3-20*01 | IGKJ1*01 |  |
| 4 | 15910 | GFTFSSYC | IKQDGSEK | AREAFLEWLAPLGYYYMDV | IGHV3-7*03 | IGHD3-3*02 | IGHJ6*03 | QSVSSSY | GAS | QQNGRSPRT | IGKV3-20*01 | IGKJ1*01 |  |
| 4 | 15964 | GFTFSTSW | IKQDGSEK | AREAFLEWLAPLGHYYMDV | IGHV3-7*03 | IGHD3-3*02 | IGHJ6*03 | QSVSSSY | GAS | QLYGRSFRT | IGKV3-20*01 | IGKJ1*01 |  |
| 5 | 15956 | GYTFTSYY | INPSGGST | ARDRYPTVVKYFGMDV | IGHV1-46*01 | IGHD4-23*01 | IGHJ6*02 | QSVSGY | DAS | QQRSNWPPSIT | IGKV3-11*01 | IGKJ3*01 |  |
| 5 | 15974 | GFTFTTYY | INPSGGST | ARDRYPTVTKYFGMDV | IGHV1-46*01 | IGHD4-17*01 | IGHJ6*02 | QSVSGY | DAS | QQRSNWPPSIT | IGKV3-11*01 | IGKJ5*01 |  |
| 5 | 15758 | GYTFTTYY | INPSGGST | ARDRYPTVVNYFGMDV | IGHV1-46*01 | IGHD4-23*01 | IGHJ6*02 | QSVSGY | DTS | QQRSNWPPSIT | IGKV3-11*01 | IGKJ5*01 |  |
| 6 | 15791 | GFTFSFYS | ISSSSRYI | ARGDDFLVVAGTIDYSHYVMDV | IGHV3-21*01 | IGHD2-2*02 | IGHJ6*02 | QNLTRW | DAS | QQYNSYPRT | IGKV1-5*01 | IGKJ2*02 |  |
| 6 | 16042 | GFTFSFYS | ISSSSRYI | ARGDDFVVVAGTIDYSHYVMDV | IGHV3-21*01 | IGHD2-2*03 | IGHJ6*02 | QNLTRW | DAS | QQYNSYPRT | IGKV1-5*01 | IGKJ2*01 |  |
| 7 | 15941 | GYTFTGYY | INPNSGGT | ARVNHYDSSGYYGWFDP | IGHV1-2*02 | IGHD3-22*01 | IGHJ5*02 | QSVLYSSNNKNY | WAS | QQYYTTPYT | IGKV4-1*01 | IGKJ2*01 |  |
| 7 | 15845 | GYTFTGYY | INPNSGGT | ARVNYYDSSGYYGWFDP | IGHV1-2*02 | IGHD3-22*01 | IGHJ5*02 | QSVLYSSNNKNY | WAS | QQYYSTPYT | IGKV4-1*01 | IGKJ2*01 |  |
| 8 | 16038 | GGSISSGDYY | IYYSGNT | ARGPKSLSGIAMAGAFDY | IGHV4-30-4*01 | IGHD6-19*01 | IGHJ4*02 | QSISSW | DAS | QQFNSYP | IGKV1-5*01 | IGKJ2*03\| IGKJ2*04 |  |
| 8 | 16028 | GGSISSGDYY | IYYSGST | ARGPKSLAGIAMTGAFDY | IGHV4-30-4*01 | IGHD6-19*01 | IGHJ4*02 | QSIRNW | DAS | QQYNTYS | IGKV1-5*01 | IGKJ2*03\| IGKJ2*04 |  |
| 9 | 15954 | GYTFTNYY | INPGGGST | RRSGWSVTDYFDY | IGHV1-46*01 | IGHD6-19*01 | IGHJ4*02 | QSLLDSDDGNTY | MLS | MQRIELPHT | IGKV2-40*01\| IGKV2D-40*01 | IGKJ5*01 |  |
| 9 | 15777 | GYSFTSYY | INPSGGST | RRSGWSVTDYFDY | IGHV1-46*01 | IGHD6-19*01 | IGHJ4*02 | QTLFDSDDGNTY | TLS | MQRLEFPHT | IGKV2-40*01\| IGKV2D-40*01 | IGKJ2*01 |  |
| 10 | 15951 | GYTFTNYY | INPGSGGT | VVRYFDWNGPFDY | IGHV1/  OR15-5*02 | IGHD3-9*01 | IGHJ4*02 | QGISNY | DAS | QKYNSVPWT | IGKV1-27*01 | IGKJ1*01 |  |
| 10 | 15966 | GYTFTNYY | INPHSGGT | VVRYFDWNGPIDY | IGHV1/  OR15-1*04 | IGHD3-9*01 | IGHJ4*02 | QGISNY | AAS | QKYDSAPWT | IGKV1-27*01 | IGKJ1*01 |  |

**References**

1. Lee DW, Khavrutskii IV, Wallqvist A, Bavari S, Cooper CL, Chaudhury S. BRILIA: integrated tool for high-throughput annotation and lineage tree assembly of B-cell repertoires. Front Immunol. 2016;7:681.
