# Supplementary material for: Using the antibody-antigen binding interface to train image-based deep neural networks for antibody-epitope classification
Source: PLoS Comput Biol. 2021 Mar 29;17(3):e1008864. doi: 10.1371/journal.pcbi.1008864 (PMC8032195; doi:10.1371/journal.pcbi.1008864)
Supplement: S3 Table — Abs in Set1 and Set2 bind to the GP1 base. Abs in Set3 target the α-helical heptad repeat 2 in the GP2 “stalk” (HR2) region. The ID numbers correspond to the Abs described in reference [23]. (DOCX) [file pcbi.1008864.s006.docx]

S3 Table. *Sets of anti EBOV antibodies used for DNN training and testing for epitope recognition*.

Abs in *Set_1_* and *Set_2_* bind to the GP1 base. Abs in *Set_3_* target the α-helical heptad repeat 2 in the GP2 ‘‘stalk’’ (HR2) region. The ID numbers correspond to the Abs described in reference [1].

| *Set_1_* | *Set_2_* | *Set_3_* |
| --- | --- | --- |
| 15734 | 15742 | 15732 |
| 15743 | 15759 | 15785 |
| 15783 | 15847 | 15811 |
| 15839 | 15850 | 15819 |
| 15843 | 15878 | 15820 |
| 15861 | 15879 | 15841 |
| 15877 |  | 15848 |
| 15908 |  | 15852 |
| 15947 |  | 15901 |
|  |  | 15925 |
|  |  | 15941 |
|  |  | 15963 |
|  |  | 15974 |
|  |  | 15975 |
|  |  | 16062 |

**References**

1. Bornholdt ZA, Turner HL, Murin CD, Li W, Sok D, Souders CA, et al. Isolation of potent neutralizing antibodies from a survivor of the 2014 Ebola virus outbreak. Science. 2016;351(6277):1078-83.
