# Supplementary material for: Using the antibody-antigen binding interface to train image-based deep neural networks for antibody-epitope classification
Source: PLoS Comput Biol. 2021 Mar 29;17(3):e1008864. doi: 10.1371/journal.pcbi.1008864 (PMC8032195; doi:10.1371/journal.pcbi.1008864)
Supplement: S4 Table — Summary of 40 DNN models used for classification of 28 antibodies belonging to ten family lineages using fingerprints colored using the reduced amino acid alphabet color-coding. (DOCX) [file pcbi.1008864.s007.docx]

S4 Table. *Detection of lineage family*.

Summary of 40 DNN models used for classification of 28 antibodies belonging to ten family lineages using fingerprints colored using the reduced amino acid alphabet color-coding.

| Lineage | Precision local | Recall local | F1-score local | Support |
| --- | --- | --- | --- | --- |
| 1 | 0.98 | 0.16 | 0.27 | 8000 |
| 2 | 0.94 | 0.33 | 0.49 | 8000 |
| 3 | 0.95 | 0.76 | 0.84 | 8000 |
| 4 | 0.89 | 0.54 | 0.67 | 8000 |
| 5 | 0.52 | 0.51 | 0.52 | 8000 |
| 6 | 0.72 | 0.80 | 0.76 | 8000 |
| 7 | 0.47 | 0.69 | 0.56 | 8000 |
| 8 | 0.47 | 0.92 | 0.62 | 8000 |
| 9 | 0.36 | 0.53 | 0.43 | 8000 |
| 10 | 0.41 | 0.45 | 0.43 | 8000 |
|  |  |  |  |  |
| micro average | 0.57 | 0.57 | 0.57 | 80000 |
| macro average | 0.56 | 0.47 | 0.47 | 80000 |
| weighted average | 0.67 | 0.57 | 0.56 | 80000 |
|  |  |  |  |  |
| Correct predictions: | 45448 |  |  |  |
| Total predictions: | 80000 |  |  |  |
| Global accuracy:^a^ | 0.57 |  |  |  |

^a^ Values computed with the Python Scikit-learn library for machine learning and statistical modeling [1].

**References**

1. Pedregosa F, Varoquaux G, Gramfort A, Michel V, Thirion B, Grisel O, et al. Scikit-learn: machine learning in Python. J Mach Learn Res. 2011;12:2825-30.
