# Supplementary material for: Using the antibody-antigen binding interface to train image-based deep neural networks for antibody-epitope classification
Source: PLoS Comput Biol. 2021 Mar 29;17(3):e1008864. doi: 10.1371/journal.pcbi.1008864 (PMC8032195; doi:10.1371/journal.pcbi.1008864)
Supplement: S6 Table — Sequence annotations of Abs from the normal class included in the training set. The sequence analysis was carried out using BRILIA [24]. (DOCX) [file pcbi.1008864.s009.docx]

S6 Table: *Detection of Abs from diverse clonotypes using the RCAE method.* Sequence annotations of Abs from the normal class included in the training set. The sequence analysis was carried out using BRILIA [1].

| **Training set** | | | | | | | | | | | |
| --- | --- | --- | --- | --- | --- | --- | --- | --- | --- | --- | --- |
| Ab Id | CDRH1 | CDRH2 | CDRH3 | VH Germline | DH Germline | JH Germline | CDRL1 | CDRL2 | CDRL3 | VL Germline | JL Germline |
| 16031 | GGSFSGYY | INHSGST | ARALPRGGNYRTFDY | IGHV4-34*01 | IGHD1-7*01 | IGHJ4*02 | QSLLHSNRYNY | MGS | MQGLQTPLT | IGKV2-28*01\| IGKV2D-28*01 | IGKJ4*01 |
| 15977 | GGSFSGYY | INHSGST | ARALPRGGNYRTFDY | IGHV4-34*01 | IGHD1-7*01 | IGHJ4*02 | QSLLHSNGYNY | MGS | MQGLQTPLT | IGKV2-28*01\| IGKV2D-28*01 | IGKJ4*01 |
| 15734 | GGSlSGYY | ItHSGST | ARGKSSTVVSRTTHFDY | IGHV4-34*01 | IGHD4-23*01 | IGHJ4*02 | QSLLHSNAYNY | MRS | MQALQAP | IGKV2-28*01\| IGKV2D-28*01 | IGKJ2*02 |
| 15947 | GGSFSGHY | INHSRRS | ARGRSSTVVSRTTHFDY | IGHV4-34*01 | IGHD4-23*01 | IGHJ4*02 | QSLLHSNGYNT | MAS | MQAQQTPIT | IGKV2-28*01\| IGKV2D-28*01 | IGKJ3*01 |
| 15843 | GGSFSGYS | INHSGST | ARAWLRSRGYPSFDY | IGHV4-34*01 | IGHD6-13*01 | IGHJ4*02 | QSLLHSNGYNY | MGS | MQALQTLT | IGKV2-28*01\| IGKV2D-28*01 | IGKJ5*01 |
| 15978 | GGSFSGYY | INHSGST | ARAWLRTSWYPSFDY | IGHV4-34*01 | IGHD6-13*01 | IGHJ4*02 | QSLLHSNGYNY | MGS | MQALQTLT | IGKV2-28*01\| IGKV2D-28*01 | IGKJ5*01 |
| 15861 | GGSFSGYH | INHSGST | ARAWLRSSSYPSFDY | IGHV4-34*01 | IGHD6-13*01 | IGHJ4*02 | QSLLHSNGYNY | MGS | MQALQTLT | IGKV2-28*01\| IGKV2D-28*01 | IGKJ5*01 |
| 15908 | GGSFSGYY | INHSGST | ARRLQRHGNYVGSFDY | IGHV4-34*01 | rIGHD2/OR15-2a*01\|rIGHD2/  OR15-2b*01 | IGHJ4*02 | QSLLHNNGYNY | MGS | MQALQAPVT | IGKV2-28*01\| IGKV2D-28*01 | IGKJ5*01 |
| 15839 | GGSFSGYY | INHSGST | ARALPRGGNYRTFEY | IGHV4-34*01 | IGHD1-26*01 | IGHJ5*02 | QSLLHSNGYNY | MGS | MQGLQTPLT | IGKV2-28*01\| IGKV2D-28*01 | IGKJ4*01 |
| 15943 | GGSFSAYY | INHSGST | ARHAHSGMDV | IGHV4-34*01 | rIGHD1-26*01 | IGHJ6*02 | QSLLHSNGYNY | MGS | MQALQPPIT | IGKV2-28*01\| IGKV2D-28*01 | IGKJ5*01 |
| 15783 | GGSFSGYY | INHSGST | ARGRRNIWLVPNYSVDV | IGHV4-34*01 | IGHD2/OR15-2a*01\|IGHD2/  OR15-2b*01 | IGHJ6*03 | QSLLHSNGYKY | MGS | MQTLQTPRT | IGKV2-28*01\| IGKV2D-28*01 | IGKJ2*01 |
| **Antibodies detected** | | | | | | | | | | | |
| Ab Id | CDRH1 | CDRH2 | CDRH3 | VH Germline | DH Germline | JH Germline | CDRL1 | CDRL2 | CDRL3 | VL Germline | JL Germline |
| 15877 | GVSISSYY | IYYTGST | ARAAKGCTSTSCYEWDV | IGHV4-59*01 | IGHD2-2*01\| IGHD2-2*03 | IGHJ6*04 | QSLLHSNGYNY | MGS | MQALQTPLT | IGKV2-28*01\| IGKV2D-28*01 | IGKJ3*01 |
| 15958 | GGSFSGYY | ISLSGST | ARVSLYFGYYMDV | IGHV4-34*01 | rIGHD3-10*01 | IGHJ6*03 | QSVSSSY | GAS | QQYGSSSLVT | IGKV3-20*01 | IGKJ4*01 |
| 16005 | GGSFSGYY | INHSGYT | VRGPPGWYSSDWPIDY | IGHV4-34*05 | IGHD6-19*01 | IGHJ4*02 | QDIRNY | DAF | QQYDNLPRT | IGKV1-33*01\| IGKV1D-33*01 | IGKJ3*01 |
| 15741 | GVSISSYY | IFTGGST | ARDPGRGRDYYEMDV | IGHV4-4*07 | rIGHD2-2*01\| rIGHD2-2*02\| rIGHD2-2*03 | IGHJ4*03 | QSLLHSNGYNY | MGS | MQALQTPA | IGKV2-28*01\| IGKV2D-28*01 | IGKJ1*01 |
| A15935 | GYTFRNYG | ISAYNGYT | ARDLTPRYGLDV | IGHV1-18*04 | IGHD3-9*01 | IGHJ6*02 | QSLLHSNGYNY | MGS | MQALQSPWT | IGKV2-28*01\| IGKV2D-28*01 | IGKJ2*02 |
| A15952 | GGSFSDDY | INYSGST | TKVGSWNVGGMAFHI | IGHV4-34*01 | IGHD1-1*01\| IGHD1-20*01 | IGHJ3*02 | QSVSSY | EAS | QQRSHWPPRIT | IGKV3-11*01 | IGKJ5*01 |

**References**

1. Lee DW, Khavrutskii IV, Wallqvist A, Bavari S, Cooper CL, Chaudhury S. BRILIA: integrated tool for high-throughput annotation and lineage tree assembly of B-cell repertoires. Front Immunol. 2016;7:681.
