# Supplementary material for: Using the antibody-antigen binding interface to train image-based deep neural networks for antibody-epitope classification
Source: PLoS Comput Biol. 2021 Mar 29;17(3):e1008864. doi: 10.1371/journal.pcbi.1008864 (PMC8032195; doi:10.1371/journal.pcbi.1008864)
Supplement: S7 Table — Statistical summary of the 10 top DNN models trained on anti EBOV Abs from a single-family lineage, and used to distinguish other Abs from the same competition group. (DOCX) [file pcbi.1008864.s010.docx]

S7 Table: *Detection of Abs from the same EBOV competition group using the RCAE method.*

Statistical summary of the 10 top DNN models trained on anti EBOV Abs from a single-family lineage, and used to distinguish other Abs from the same competition group.

| DNN model | Training and Validation Image Set Sizes | | | Testing Image Set & Results | | |  |
| --- | --- | --- | --- | --- | --- | --- | --- |
|  | N_training_^a^ | M_validation_^b^ | | N_test_^c^ | | AUROC Test^d^ | normal Ab Id^e^ |
|  | *normal* | *normal* | *anomalous* | *normal* | *anomalous* |  |  |
| 1 | 986 | 174 | 12700 | 100 | 5200 | 1.00 | **A15877** |
| 2 | 1122 | 198 | 12100 | 100 | 5800 | 0.99 | **A15958** |
| 3 | 986 | 174 | 12300 | 100 | 5600 | 0.99 | **A16005** |
| 4 | 1020 | 180 | 12100 | 100 | 5800 | 0.99 | **A15741** |
| 5 | 1122 | 198 | 12100 | 100 | 5800 | 0.97 | **A15935** |
| 6 | 1122 | 198 | 12700 | 100 | 5200 | 0.96 | **A15952** |
| 7^f^ | 1020 | 180 | 12700 | 100 | 5100 | 0.96 | A15886 |
| 8 | 1020 | 180 | 12100 | 100 | 5800 | 0.96 | **A15865** |
| 9^f^ | 233 | 127 | 12600 | 100 | 5200 | 0.96 | A15925 |
| 10^f^ | 233 | 127 | 12900 | 100 | 4900 | 0.94 | A15820 |
|  |  |  |  |  |  |  |  |

^a^ N_training_; number of fingerprints from normal Abs selected for training of the model.

^b^ M_validation_; number of fingerprints from normal and anomalous Ab classes selected for validation.

*^c^* N_test_; number of fingerprints from normal and anomalous Ab classes in the testing sets.

^d^ AUROC Test is computed on the testing set using the Python Scikit-learn library for machine learning and statistical modeling [1].

^e^ Identification of the normal Ab used for testing. Abs that compete with KZ52 are highlighted in boldface.

^f^ The test set used to evaluate this DNN model contains only Abs that do not compete with KZ52 to detect false positives (i.e., the Ab representing the normal class was a decoy).

**References**

1. Pedregosa F, Varoquaux G, Gramfort A, Michel V, Thirion B, Grisel O, et al. Scikit-learn: machine learning in Python. J Mach Learn Res. 2011;12:2825-30.
