# Supplementary material for: Using the antibody-antigen binding interface to train image-based deep neural networks for antibody-epitope classification
Source: PLoS Comput Biol. 2021 Mar 29;17(3):e1008864. doi: 10.1371/journal.pcbi.1008864 (PMC8032195; doi:10.1371/journal.pcbi.1008864)
Supplement: S8 Table — (DOCX) [file pcbi.1008864.s011.docx]

S8 Table: *Sequence comparisons of the Abs detected by the RCAE method with Abs known to bind to the base of EBOV GP_1,2_.^a^*

| Abs^b^ | 15877^c^ | 15958^c^ | 16005^c^ | 15741^c^ | 15935^c^ | 15952^c^ |
| --- | --- | --- | --- | --- | --- | --- |
| KZ52 [1] | 67 | 69 | 61 | 68 | 65 | 63 |
| C2G4 [2] | 48 | 50 | 53 | 49 | 50 | 52 |
| C4G7 [2] | 48 | 49 | 54 | 49 | 53 | 50 |
| A15734 [3] | 82 | 72 | 69 | 81 | 66 | 68 |
| A15878 [4] | 51 | 62 | 63 | 53 | 55 | 58 |
| Ab100 [5] | 71 | 62 | 54 | 71 | 41 | 59 |
| 4M0368 [6] | 61 | 63 | 81 | 62 | 56 | 60 |
| CA45 [7] | 61 | 63 | 58 | 60 | 57 | 57 |
| 6D6 [8] | 44 | 61 | 65 | 54 | 53 | 62 |
| A15946 [9] | 52 | 63 | 78 | 45 | 45 | 65 |
| A15762^d^ [3] | 51 | 56 | 62 | 50 | 50 | 57 |

^a^ The values reported in the Table correspond to the percentage of sequence identity obtained with the program BLAST [10]. For this analysis, we concatenated the sequences of the light and heavy chains of the FABs, and carried out pairwise alignments.

^b^ This column displays the Ab name and a reference to the corresponding experimental information.

^c^ The ID numbers correspond to the Abs described in reference [3].

^d^ Antibody ADI-15762 binds to the glycan cap in a shallow angle interfering with binding of KZ52.

**References**

1. Lee JE, Fusco ML, Hessell AJ, Oswald WB, Burton DR, Saphire EO. Structure of the Ebola virus glycoprotein bound to an antibody from a human survivor. Nature. 2008;454(7201):177-82.

2. Pallesen J, Murin CD, de Val N, Cottrell CA, Hastie KM, Turner HL, et al. Structures of Ebola virus GP and sGP in complex with therapeutic antibodies. Nat Microbiol. 2016;1(9):16128.

3. Bornholdt ZA, Turner HL, Murin CD, Li W, Sok D, Souders CA, et al. Isolation of potent neutralizing antibodies from a survivor of the 2014 Ebola virus outbreak. Science. 2016;351(6277):1078-83.

4. Murin CD, Bruhn JF, Bornholdt ZA, Copps J, Stanfield R, Ward AB. Structural Basis of Pan-Ebolavirus Neutralization by an Antibody Targeting the Glycoprotein Fusion Loop. Cell Rep. 2018;24(10):2723-32.e4.

5. Misasi J, Gilman MS, Kanekiyo M, Gui M, Cagigi A, Mulangu S, et al. Structural and molecular basis for Ebola virus neutralization by protective human antibodies. Science. 2016;351(6279):1343-6.

6. Ehrhardt SA, Zehner M, Krähling V, Cohen-Dvashi H, Kreer C, Elad N, et al. Polyclonal and convergent antibody response to Ebola virus vaccine rVSV-ZEBOV. Nat Med. 2019;25(10):1589-600.

7. Janus BM, van Dyk N, Zhao X, Howell KA, Soto C, Aman MJ, et al. Structural basis for broad neutralization of ebolaviruses by an antibody targeting the glycoprotein fusion loop. Nat Commun. 2018;9(1):3934.

8. Milligan JC, Parekh DV, Fuller KM, Igarashi M, Takada A, Saphire EO. Structural Characterization of Pan-Ebolavirus Antibody 6D6 Targeting the Fusion Peptide of the Surface Glycoprotein. J Infect Dis. 2019;219(3):415-9.

9. West BR, Wec AZ, Moyer CL, Fusco ML, Ilinykh PA, Huang K, et al. Structural basis of broad ebolavirus neutralization by a human survivor antibody. Nat Struct Mol Biol. 2019;26(3):204-12.

10. Altschul SF, Gish W, Miller W, Myers EW, Lipman DJ. Basic local alignment search tool. J Mol Biol. 1990;215(3):403-10.
