# Supplementary material for: Using the antibody-antigen binding interface to train image-based deep neural networks for antibody-epitope classification
Source: PLoS Comput Biol. 2021 Mar 29;17(3):e1008864. doi: 10.1371/journal.pcbi.1008864 (PMC8032195; doi:10.1371/journal.pcbi.1008864)
Supplement: S9 Table — Images were obtained from Wikimedia Commons (commons.wikimedia.org) & Creative Commons (creativecommons.org) (DOCX) [file pcbi.1008864.s012.docx]

S9 Table. *List of cat and dog images used to produce S1 Fig.*

Images were obtained from Wikimedia Commons (commons.wikimedia.org) & Creative Commons (creativecommons.org)

| **Image Source** | **Author** | **Licensed under** |
| --- | --- | --- |
| <https://search.creativecommons.org/photos/59d0a647-6945-44ca-9838-fde66e581896> | Jelly Dude | "Shortly dude" by Jelly Dude is licensed with CC BY 2.0.*^a^* |
| <https://commons.wikimedia.org/wiki/File:German-shepherd-4040871920.jpg> | Hans Kemperman | This file is made available under the Creative Commons CC0 1.0 Universal Public Domain Dedication.*^b^* |
| <https://commons.wikimedia.org/w/index.php?title=File:Doberman-12833551920.jpg&oldid=460491768> | YamaBSM | This file is made available under the Creative Commons CC0 1.0 Universal Public Domain Dedication.*^b^* |
| <https://search.creativecommons.org/photos/6311e3f7-5533-4cb7-9e54-9f71c8ef2c67> | sabianmaggy | "Maggy Cat" by sabianmaggy is licensed with CC BY 2.0.*^a^* |
| <https://search.creativecommons.org/photos/74c29036-8156-4251-ade6-7ed6a437c21d> | Robert Couse-Baker | "2014-027 - clingly cat" by Robert Couse-Baker is licensed with CC BY 2.0.*^a^* |
| <https://search.creativecommons.org/photos/1266e61c-be5d-457b-b459-c2b04ce23480> | eva101 | "Cat Mandoo too" by eva101 is licensed with CC BY 2.0.*^a^* |

*^a^* To view a copy of this license, visit https://creativecommons.org/licenses/by/2.0/

^b^ To view a copy of this license, visit https://creativecommons.org/publicdomain/zero/1.0/
