# Supplementary material for: Using the antibody-antigen binding interface to train image-based deep neural networks for antibody-epitope classification
Source: PLoS Comput Biol. 2021 Mar 29;17(3):e1008864. doi: 10.1371/journal.pcbi.1008864 (PMC8032195; doi:10.1371/journal.pcbi.1008864)
Supplement: Attachment — Submitted filename: Response-to-Reviewers-FEB0521.docx [file pcbi.1008864.s013.docx]

**Editor Comments:**

Charlotte M Deane
Associate Editor
PLOS Computational Biology

Nir Ben-Tal
Deputy Editor
PLOS Computational Biology

The reviewers have raised significant concerns with the paper in particular whether the method offers any real advantages over much faster sequence based methods which must be fully addressed in any revised submission.

We appreciate the enthusiasm and detailed comments of the Reviewers, but at the same time we would also like to point out there are several aspects of introducing a new methodology that may not be easily covered in a single journal article. The aim here is to describe the feasibility and backbone of the technology, not necessarily to show that this technology can outperform all existing technologies in all aspects of Abs recognition. Some aspect of the Review raise significant concerns that can by necessity only be partially answered here, but we have addressed the major concerns raised. In particular, we removed Ab duplicates with identical CDR sequences in our analysis of lineage detection and repeated all calculations affected by the replacement. We obtained similar results as those from our previous analyses, which did not alter our main conclusions. Secondly, we added materials to ascertain Ab detection of non-sequence similar Ab competitors as a final validation of the technology to identify specific Abs from a larger set of Abs.

In summary, we have addressed the major issues raised and hope that the manuscript will be of interest and value to the PLOS CB community.

**Editorial Office Comments:**

We have corrected the ms. submission according to the Editorial Office Comments. Please note that we removed the reference to “data not shown” on page 10, line 265, and reported only the average accuracy and kappa values obtained for the particular calculations without image enhancement. We have also added on page 52 a list of legends for the supporting Information as instructed. Lastly, we have modified Fig.S1 and Table S9 in the supporting Information to include images of dogs and cats licensed under a CC-BY 2.0 and CC0 1.0 licenses

**Comments to the Authors:**

**Reviewer #1:**

Ripoll et al. present a novel machine learning method trained on in vitro-confirmed epitope binders to classify antibody binding sites by their likely epitope engagement. Their goal is that this method could be applied to high-throughput sequencing (HTS) datasets to more reliably identify the subset of antibodies that bind to a particular antigen epitope. The current state-of-the-art, given a set of known binders to an epitope, is to search through HTS datasets for antibodies of very close genetic relatedness (same closest V/J genes and high CDRH3 sequence identity, `clonotyping’) and to shortlist these as likely to be functionally equivalent. Ripoll et al.’s proposed method is compelling, as it goes beyond a purely sequence-based approach by taking as input flattened 2D representations of 3D-models of each antibody binding site; such explicit topological consideration could therefore identify genetically-similar antibodies that have too different a topology to bind to the same epitope, reducing false positives. Their proposed framework also has the potential, if trained on a sufficiently diverse set of clonally distinct antibodies that can bind to the same binding site, to capture the crucial topological and chemical features that govern epitope complementarity, and thus to be able to cluster more diverse antibodies than clonotyping with likely functional commonality. Proof of either of these two advances would represent a significant step forward for the field.

The Reviewer has articulated the ultimate goal of this research very well and our manuscript describes the foundations for this effort. This means that we have spent considerable time evaluating the basic premise of the approach and tested the methodology in examples that are necessary, but may not be perceived as the most exciting examples where the possible future benefit is the highest. This is why we have selected the Methodology Paper track as most appropriate for this manuscript. It will not be possible to create a formal proof of the described approach – we are restricted to using known examples from the literature that may or may not contain all the information needed to full test all premises of the method. We are striving to give a complete picture of the method foundations – notice that not all examples are perfectly scored indicating that there is potentially room for improvements or understanding restrictions in applications where the propose method will not work.

As the manuscript is currently constructed, it is impossible to say whether their DNN architecture is sufficiently sensitive to detect the former category (i.e. discern between sequence-similar antibodies that can/cannot engage the same epitope).

The only antibodies used in training are ones that are proven to bind to one of multiple epitopes on an antigen's surface, and each epitope’s binders appear to be highly genetically distinct (judging by the EBOV binders to 3 epitopes, sourced from GenBank via. Bornholdt et al.).

The algorithm would need to see sequence-similar negative examples, i.e. antibodies that are close in sequence to known binders but whose binding capabilities are destroyed by particular mutations/the use of significantly different CDR3s.

To address the last point first, our method is not able to determine if a single mutation can disrupt binding. The precise role of a single mutation is very difficult to predict using any method, even in the most sophisticated perturbation calculation of free energy changes requires both precise knowledge of the 3-D structure of the antibody-antigen complex as well as an inordinate amount of computer time.

Instead, the objective of the work was to show that the methodology – at its current stage of development – can successfully distinguishing Abs that recognize different epitopes (without precise knowledge of the epitopes themselves) based on patterns derived from the spatial distribution of charged residues in the paratope. The DNN models do not explicitly use sequences of Abs to generate predictions, instead we use the spatial distribution of charged residues (colored blue and red) in a background of polar and nonpolar type of residues (all colored in shades of grey). Thus, any mutation that does not disrupt this patter in a major fashion will not be captured in our approach, e.g., such as a Val/Ala mutation. To clarify this point, we included the following additional text in the manuscript (page 29, lines 847-849) in the Discussion:

“*It must be noted that the current method is not intended to address problems such as the prediction of single mutation that disrupt binding or destabilize Ab-antigen complexes.*”

We also expanded on this in the sub-Section *Generation of fingerprints* in the *Materials and Methods* (page 39, lines 1137-1142):

*“It is worth noting that the amount of information contained in the sequence and the structural model of an Ab is substantially reduced in the fingerprints. There is no residue-connectivity information and, in particular, for fingerprints colored by charge, sequence and structural information of the Ab is reduced to a two-dimensional map of the Ab binding site based on a 3-color representation of the residues.”*

It is possible that the method can detect clonally distinct antibodies that can bind to the same epitope, but it is not clear from how the manuscript is currently presented and may depend on the tested epitope. EBOV epitope 1, for example, is so homogenous in its binding examples that the DNN could learn trivial classification features (11/12 binders belong to the IGHV4-34/IGHJ4 germline, of which 9 have at least one partner from the same VH clonotype [see example]; and no other epitope has an antibody from the IGHV4-34 germline that can bind to it). Atomic patterns of the IGHV4-34 CDRH1/CDRH2 alone could therefore dominate the classification parameters and may in practise lead to any IGHV4-34-derived antibody that is fed into the model being predicted as an Epitope 1 binder (highly unlikely to be meaningful when applied to HTS). Relatedly, it seems strange that so many tables (3, 9, 10) describe the results of applying the DNN to detect antibody lineages. As the authors say, this is already trivial by sequence, and in my view the exciting feature of this methodology is that it has the potential to see beyond lineage to tell researchers orthogonal information about which chemical/topological features are necessary to bind to a particular epitope.

EXAMPLE
ADI-15843: IGHV4-34 + IGHJ4 + ARAWLRSRGYPSFDY + IGKV2-28 + IGKJ5 + MQALQTLT
ADI-15912: IGHV4-34 + IGHJ4 + ARAWLRSRGYPSFDY + IGKV2-28 + IGKJ5 + MQALQTLT
These two antibodies to the same EBOV epitope differ by just 3 amino acid residues across the entire Fv. Fingerprints of an identical VJH3 + VJL3 lineage are therefore currently allowed to co-exist in the training and test set.

We have critically examined our work in light of these comments and focused our main revision effort in examining and expanding on Abs recognition using examples from different lineages as detailed below.

We removed Ab-duplicates with identical CDR sequences. The sequences that we used for the HIV experiments contained no duplicates. Thus, we did not introduced changes in the manuscript related to the HIV application. With respect to the sections involving antibodies against Ebola, we replaced ADI-15912 with ADI-15908, since this Ab shares the HV, JH, VL, and JL germlines with other members of this family, broadening the definition of lineage family. Then, we repeated all calculations affected by the replacement of ADI-15912 with ADI-15908. We obtained similar results as those from our previous analyses, which did not alter our main conclusions. We modified the text and Tables of the manuscript to incorporate these changes. We added a Supplemental Table S2 listing the Ab lineages, and the CDR sequences, highlighting the charged residues.

On pages 11-14, lines 311-384, we changed Tables 3 and 4, and Figures 4 and 5 of sub-Section “*DNN prediction of family lineage from fingerprints of anti-EBOV Abs*,” and modified the text in the manuscript to account for those changes.

On pages 15-18, lines 418-490, we changed Table 5, 6, 7, and 8 of sub-Section “*Detection of binding site preferences of anti-EBOV GP antibodies,”* and modified the text in the manuscript to account for those changes.

On pages 20-23, lines 575-637, we changed Table 10 and Table 11 of sub-Section “*Using one-class classification methods for Ab detection,”* and modified the text in the manuscript to account for those changes.

On page 33, line 938, we updated Fig 12.

I would like to see:
(A) evidence (e.g. through LIME) that the most important features used to distinguish binders to every epitope are spread in a meaningful way throughout the CDRs.

To address this point, we used the LIME program to analyze results of a DNN model trained to predict the specificity of EBOV Abs for three possible sites. For this analysis, we selected a group of fingerprints from the three Ab classes that the trained DNN model predicted correctly. On pages 26-27, lines 756-802, in sub-Section “*Explanation of DNN classifications using an interpretable model*,” we replaced the results of our previous example with a description of the new LIME analysis, and its main findings. We added Fig 10 to show that the main features used for recognition are consistently similar for fingerprints of a given class. The figure also shows that areas of the fingerprints associated with these important features detected by the DNN model are markedly different among fingerprints from the three Ab classes.

(B) evidence that good model performance is not simply dependent on testing on an example with a same-VH clonotype binder in the training set (test accuracy seems to vary widely depending on the training/testing split). This could be provided by not only blinding the model at training-time to fingerprints from the tested antibody, but also to those of same-epitope binding antibodies from the same clonotype/lineage. LIME could also be used here to look at how the important features change based on this blinding procedure. If the model cannot perform well without knowledge of a same-lineage binder, it would offer little more practical value than clonotyping (if I already knew an antibody from that lineage bound that epitope, then clonotype “fishing” can currently highlight antibodies from the same lineage as worthy of investigation).

We were not able to find a sufficiently large number of Abs from different clonotypes that target the same epitope in PDB or the CryoEM database; most of the antibodies that bind to the same epitope in EBOV GP and HIV GP120/GP41 belong to the same lineage. Thus, if we reduce our sets to one representative per lineage, we are left with very few Abs and not enough diversity to carry out meaningful computational experiments.

We want to raise a point regarding the Reviewer request, and explain why we still believe that our tests are valid. Note that the fingerprints do not carry explicit sequence information or residue connectivity and the DNNs are not fed with separate sequence information either. Probably, a fair assessment of our method is to compare its results with that of a sequence analysis where all non-charged residues are blinded, and only three types of residues are used, e.g., as in using only A [non-charged], [positive] P, and N [negative] in a sequence such as …AAAAAAPAAAANAAAAA... Three-letter sequence alphabets cannot reliably detect family relations based solely on information on the distribution of charged residues in the sequence.

Although we could not carry out the test proposed by the Reviewer in the studies we presented, we addressed the critique by adding an additional OCC experiment that avoids using sequences from the same lineage simultaneously in the training and test sets. We explained this study in detail in the response to the next point raised by the Reviewer.

(C) An OCC experiment trained with the "normal class" of antibodies that can bind the same epitope (again blinded to antibodies from the same lineage as the tested antibody), rather than antibodies deriving from the same lineage family, to see whether the model can provide more insight than would be provided by sequence-based lineage clustering alone.

To address these core criticisms, we carried out additional tests using the OCC method and imposing the conditions requested by the Reviewer. On this basis, we selected as the normal class, Abs from a family lineage that recognize a particular epitope on EBOV GP and compete with Ab KZ52 for binding. This set of Abs, together with the anomalous class consisting of various Abs that do not compete with KZ52, were used to train DNNs. We tested if these DNNs could detect Abs from different clonotypes as belonging to the same class of binders.

To evaluate the trained DNN models we constructed 100 test sets with ~5,000 fingerprints (100 fingerprints per Ab) from the remaining ~50 anomalous Abs and a single Ab “tagged” as a member of the normal class. The putative normal Ab was extracted from two types of Abs: KZ52 competitors or non-competitors. If a DNN model detects with high certainty an Ab from the actual normal class, such an Ab must belong to the pool of KZ52 competitors. DNNs that detect as normal an Ab from the list of non-competitors represents failure.

The criterion for a successful detection is based on the decoding errors produced by a DNN for every fingerprint in the test set: A DNN model generates a successful detection when the decoding errors of most of the fingerprints of the Ab tagged as normal assigned the smallest values of the whole set of images.

Four of the 100 DNN models we trained satisfied our criterion of successful detection (i.e., these DNNs detected Abs as “normal” Abs from the list of KZ52 competitors), while two other DNN models reached partial detection according to the criteria we used. Importantly, none of the detected Abs were from the same family lineage of the normal training set.

Independently, we collected a series of experimental structures of anti-EBOV Abs in complex with GP_1,2_, describing the epitopes recognized by the Abs. These Abs are bound to eleven different epitopes in EBOV GP. We carried out sequence comparisons between the Abs detected by the DNNs and the eleven experimentally determined Abs, and found that Ab ADI-15734 from the latter group is the closest homolog to our candidates, based on the percentage of sequence identity. These results support the predictive results of the DNNs as ADI-15734 belongs to the set of “normal” Abs used for training. We introduced a Table S8 with the results of this sequence analysis, and we added references to the Table in the text of the manuscript.

Thus, we showed that DNNs trained with a small set of Abs with little sequence diversity detected a handful of candidates that belong to same group of KZ52 competitors as the set of “normal” Abs used for training. We also showed that DNNs produced no false-positive detection in multiple (~60) negative test cases built only with non-competitors of KZ52. The Abs detected correctly by the DNNs are likely to share the binding preference of the training set based on sequence and structural comparisons with Abs from experimentally determined complexes of EBOV GP. The detected Abs do not share the lineage of the “normal” set of Abs used in training.

The extensive description of this new experiment and its results are included within sub-Section “*Using one-class classification methods for Ab detection,*” on pages 23-28, lines 639-754. We also added a couple of sentences into the Discussion section, page 34, lines 898-902, related to this new experiment.

Other general points:
- The method is notable in the fact that it could capture the influence of the light chain CDRs on binding specificity. As most deep antibody repertoire sequencing datasets are currently heavy chain only, it would be interesting to see the effect on predictive performance if the light-chain component of the fingerprints is removed.

Our present efforts have been focused on developing the methodology for paired sequencing data to show that this approach is feasible. We have considered generating 3-D models of Abs using heavy chain sequence information and a dummy model of the light chain, and are planning to investigate this possibility in a future study.

- The authors should explain in detail the origin of the stochasticity generated by modelling the same antibody multiple times and explain why it is a good proxy for binding site flexibility. I could not find this described in the Methods section.

We extended the Method Section “*Generation of 3-D models of Abs”* and added an additional figure (Fig 13) on page 36, lines 1114-1128, to justify the use of multiple models in the search for structural patterns.

We added the following text on page 36, lines 1099-1112:

*In analogy with NMR structure determinations where the experimental structure is defined by a conformational ensemble, we used multiple 3-D models of the same Ab in an attempt to capture primarily the inherent flexibility of the CDRs that determine the Ab paratope. As an example, we show in Fig 13A the superposition of a single model for seven EBOV Abs that to recognize the stalk region of EBOV GP using a simplified representation. These models highlight charged residues located in the CDRs and show that equivalent residues in other Abs can occupy different positions in the 3-D space due to changes in the CDRs or the orientation of the side chains. Consequently, the fingerprints associated with these Abs may show very different patterns. By using multiple models for a given Abs, as shown in Fig 13B-C, we can generate multiple fingerprints with variations in their patterns, with some of them resembling closer those present in the models of other Abs of the group. By training DNNs on the multiple patterns of each Ab, we expect that the trained DNN models will be able to extract consensus features that help identifying Abs with similar characteristics.*

- Certain methodological changes (e.g. charge colour scheme/reduced amino acid alphabet and image enhancement/no image enhancement) should be discussed - why the do the authors believe the chosen representations can yield such different accuracies?

To address the Reviewer comments, we expanded the discussions on coloring schemes in “*Methodological Considerations and Limitations,*” on pages 31-32, lines 932-938:

*Intuitively, we expected that DNN training using fingerprints based on a reduced-residue code would improve the predictive capacity of the models given that such fingerprints contain additional information regarding other types of residues beyond the charge ones. While we do not have a clear explanation for the lack of improvement of the predictions, the new fingerprint color patterns due to the additional residue types contribute noise in terms of information overflow that weakens the actual signal instead of enhancing it.*

To explain further the usage of image enhancement, we added the following to the Method Section “*Training and testing DNN for antibody classification on fingerprints*” (page 38, lines 1182-1186):

*This procedure is quite useful when a reduced number of images is available for DNN training and aids in limiting overfitting. Thus, to augment the training set, new images are generated from the original ones via random transformations. Effectively, the DNN model never sees the same image more than once.*

- The authors state “comparison of the 3-D structure of every Ab in a repertoire is unfeasible”. They should clarify that this refers to experimental structure determination, as published software already exists to computationally perform full-repertoire structural comparisons (e.g. SAAB+, 10.1371/journal.pcbi.1007636)

We followed the Reviewer’s suggestions and modified the text (page 4, lines 93-94) as follows:

*Experimental determination of the 3-D structure of every Ab in a repertoire is unfeasible.*

We also added the following references to computational approaches to model structures of Abs in repertories:

6.	Kovaltsuk A, Raybould MIJ, Wong WK, Marks C, Kelm S, Snowden J, et al. Structural diversity of B-cell receptor repertoires along the B-cell differentiation axis in humans and mice. PLoS Comput Biol. 2020;16(2):e1007636.

7.	DeKosky BJ, Lungu OI, Park D, Johnson EL, Charab W, Chrysostomou C, et al. Large-scale sequence and structural comparisons of human naive and antigen-experienced antibody repertoires. Proc Natl Acad Sci U S A. 2016;113(19):E2636-45.

**Reviewer #2: Using Paratope Features to Train Deep Neural Networks for Antibody
Classification**

The authors use a very novel and clever approach to attempt to discover patterns in antibody sequence/structural motifs that allow for prediction of specificity to antigen.

While it is very clever to use 3D structural predictions of paratope combined with biophysical features to then create a 2D-image of paratope, there are questions as to whether this level of elaborative methods is necessary compared to more conventional sequence based tools.


A major question is to what extent is the 3D-structural model for Rosetta adding important information? If it is to simply model and thus define paratopes residues, then I am not convinced that it is a requisite step. Nearly all antibody paratopes are driven by CDRs, especially CDRL3 and CDRH3, and thus simply extracting CDR sequences and using them as the paratope would be a simple solution. CDRs could even be weighted as has been previously done for TCRs (see Dash, Nature, 2017).

Our main goal is to identify Abs that are not clonally related, but bind to the same region of an antigen. This is a highly non-trivial problem. Clonally unrelated Abs can engage the same region of an antigen in different orientations. In experimentally determined Abs/antigen complexes from different clonotypes, it is typically only a handful of Abs residues (from non-equivalent positions in the sequence) that are involved in creating the unique local structural arrangements that can engage with the epitope. As these structural features are the main contributor to the interaction with the antigen, a phylogenetic analysis involving only a few residues is not sufficient to detect these clonally unrelated Abs. One would need to actually determine the local structural alignment of the key residues, which cannot be directly inferred from the CDR sequences.

We approached the problem of identifying these type of antibodies using structural models that provide probable 3-D positions of residues forming the binding site. To account for the flexibility of the CDR loops, we used Rosetta Antibody to generate a series of 3-D models that are sterically possible. Rosetta Antibody generates these models using various templates that correspond to high-resolution PDB structures.

To clarify this issue, we added the following changes in the manuscript to lines 89-105 on page 4:

“*We hypothesize that the complement of residues in the Ab paratope determines the preference for a particular epitope on the antigen, and that Abs that has evolved from unrelated clonotypes to recognize this epitope, are likely to share common structural patterns and physiochemical properties even if they differ in their binding modes.*

*How can we test this hypothesis? Experimental determination of the 3-D structure of every Ab in a repertoire is unfeasible. Recent advances in computational algorithms have allowed researchers to produce structural models for thousands of Abs to complete repertoires (6, 7). Analyses of these models has led to a better understanding of the structural profiles of naïve B-cells, and the structural changes of the complementarity-determining regions (CDRs) that reshape the Ab binding sites during B-cell differentiation. However, even if we obtain a computational or experimental 3D model of the Abs, it is non-trivial to infer if two clonally distinct Abs can recognize the same or overlapping epitope(s) using different binding modes, since this task cannot be resolved using sequence based approaches. To partly overcome this problem, we can produce large conformational ensembles of the Abs employing molecular modeling techniques to account in part for uncertainties associated with the flexibility of the Ab CDRs*.”

By using a 3D model that has no certainty in accurately modeling the dynamic conformational loops of antibody CDRs only to define a slightly more minimal paratope residues does not seem like an effective strategy. Most notably it dramatically increases the time and computational power required to perform this on large sequencing datasets. If the authors are convinced about this approach, then they should directly compare the results of their classification scheme using 3D-structural models directly with simple CDR sequence based paratope definitions.

As explained above, the detection of common structural features in a pair of clonally unrelated Abs cannot be obtained from sequence analysis only.

“We note that the detection of family lineages is easily achieved with computational tools based on sequence analysis. Our objective, however, was to determine the ability of DNNs to learn to associate members of the lineage family using similarities in the image patterns based on the arrangement of color on the fingerprints.” I appreciate stating clearly that sequence analysis tools are already sufficient to cluster antibodies into family lineages and thus they simply wanted to benchmark their DNNs. But based on the data in Table 2, they achieve a global accuracy of their DNNs of 0.62. This seems extremely low when compared to simple sequence based lineage clustering. With a performance at this level the authors have to justify what value their approach really adds for lineage classification. For example, if they could classify sequences to a lineages with DNNs that fall outside of common sequence similarly metrics (e.g., 80% similarity of CDRH3) that would add value and convince me of the potential of their approach. They should thus attempt to look at this aspect.

First, the methodology we introduce does not attempt to compete with sequence-based method for lineage classification. Our main goal is to show that this approach can identify diverse sets of Abs with similar properties such as epitope preference. We use the examples of lineage recognition to show that family association can still be detected from a series of images without explicitly using sequence information. Each image or fingerprint depicts likely distribution of positively- and negatively-charged residues near the paratope region of the Ab. The explicit sequence information is not used (i.e., all non-charged residues are colored grey/white) during training of the DNNs. The results present here show that these charge patterns retain enough information to produce a meaningful association.

We believe that the additions to the manuscript on page 4, lines 94-102, can help clarify this issue:

“*Recent advances in computational algorithms have allowed researchers to produce structural models for thousands of Abs to complete repertoires (6, 7). Analyses of these models has led to a better understanding of the structural profiles of naïve B-cells, and the structural changes of the complementarity-determining regions (CDRs) that reshape the Ab binding sites during B-cell differentiation. However, even if we obtain a computational or experimental 3D model of the Abs, it is non-trivial to infer if two clonally distinct Abs can recognize the same or overlapping epitope(s) using different binding modes, since this task cannot be resolved using sequence based approaches.”*

Similar to my point above, the authors perform a classification analysis on Ebola and HIV antibodies, trying to accurately predict epitope groups. In many cases antibody sequences that bind the same epitope may have very similar germline or CDR sequences. Thus the authors should compare whether very simple machine learning classification models based on antibody sequences (germline or CDRs) could similarly predict epitope specificity. For example the aforementioned TCRdist (by Dash, Nature, 2017) was able to predict specificity of TCRs using a weighted distance-based clustering of CDR sequences followed by a k-nearest neighbor model to accurately predict TCRs specificity. Something similar could in principle work for antibodies as well and serve as an important benchmark to compare their approach.

As stated earlier, the detection of common structural features in a pair of clonally unrelated Abs cannot be obtained from sequence analysis only. The method by Dash et al., is sequence based and cannot be applied to the type problem we want to study. For this reason, we have omitted comparisons of our method with other sequence-based methods. We used the EBOV and HIV examples to show that a DNN model can still distinguish Abs classes using fingerprints with a reduced amount of information such as the spatial distribution of charged residues near the paratope, but with no sequence or residues connectivity information.

**Reviewer #3: Summary**

In this work, Ripoll and colleagues developed a paratope-based DL-classification approach. They use this approach to classify Abs belonging to, among others, different lineages. While this work is interesting, the authors fail to acknowledge prior literature and do not compare their approach to already existing approaches. The relevance of their results is thus questionable.

We modified the Introduction to include additional relevant references.

With respect to the request for comparisons with existing approaches, we note that our goal is the detection of common structural features in clonally unrelated Abs. Those features that cannot be obtained from sequence analysis only. We have not been able to find through literature searches a methodology that attempt to identify clonally distinct Abs with similar functionality.

The manuscript has a lot of redundant text. It could benefit from strict editing. Major and minor issues are written below.

We have included additional material to respond to the Reviewer comments. We made an effort to address the issues in the manuscript as succinct as possible. We eliminated the description of a previous experiment in the Results Section “Explanation of DNN classifications using an interpretable model,” on pages 26-27, lines 756-802, to include a new one that describes better the implantation of the program LIME to analyze the results of DNNs.

Major

Prior relevant literature seems to be unknown to the authors.
Just a few examples because there are too many:
“Comparison of the 3-D structure of every Ab in a repertoire is unfeasible.” → please see <https://www.frontiersin.org/articles/10.3389/fimmu.2018.01698/full> and follow-up papers.

We responded to similar issue raised by Reviewer 1. We rephrased the sentence to state that the experimental determination of all Abs in a repertoire is unfeasible. We also added references to computational methods used to generate 3-D models of complete Ab repertoires.

Page 4, line 93-102 now reads:

*“How can we test this hypothesis? Experimental determination of the 3-D structure of every Ab in a repertoire is unfeasible. Recent advances in computational algorithms have allowed researchers to produce structural models for thousands of Abs to complete repertoires (6, 7). Analyses of these models has led to a better understanding of the structural profiles of naïve B-cells, and the structural changes of the complementarity-determining regions (CDRs) that reshape the Ab binding sites during B-cell differentiation. However, even if we obtain a computational or experimental 3-D model of the Abs, it is non-trivial to infer if two clonally distinct Abs can recognize the same or overlapping epitope(s) using different binding modes, since this task cannot be resolved using sequence based approaches.”*

The added references are:

6.	Kovaltsuk A, Raybould MIJ, Wong WK, Marks C, Kelm S, Snowden J, et al. Structural diversity of B-cell receptor repertoires along the B-cell differentiation axis in humans and mice. PLoS Comput Biol. 2020;16(2):e1007636.

7.	DeKosky BJ, Lungu OI, Park D, Johnson EL, Charab W, Chrysostomou C, et al. Large-scale sequence and structural comparisons of human naive and antigen-experienced antibody repertoires. Proc Natl Acad Sci U S A. 2016;113(19):E2636-45.

“The data derived from immunological studies typically represent the result of ongoing stochastic and multifactorial processes that is often difficult to decipher. Artificial Intelligence (AI) methods are potentially well suited to address these types of problems. Thus, machine learning approaches have already been used for predicting peptide presentation by T cells (5-7), affinity of peptide binding to Major Histocompatibility Complex molecules (8), and binding affinity of neutralizing Abs (9). Deep learning techniques have also been used for de novo prediction of protein structures (10, 11).” →

I suggest the readers have a look at reviews such as: <https://academic.oup.com/bib/article/21/5/1549/5581643> or <https://www.sciencedirect.com/science/article/pii/S2452310020300524> instead of erratically citing barely relevant papers.

To address the point raised by the Reviewer, we modified the Introduction to include the suggested references.

8.	Norman RA, Ambrosetti F, Bonvin A, Colwell LJ, Kelm S, Kumar S, et al. Computational approaches to therapeutic antibody design: established methods and emerging trends. Brief Bioinform. 2020;21(5):1549-67.

9.	Greiff V, Yaari G, Cowell LG. Mining adaptive immune receptor repertoires for biological and clinical information using machine learning. Current Opinion in Systems Biology. 2020;24:109–19.

13.	Moris P, De Pauw J, Postovskaya A, Gielis S, De Neuter N, Bittremieux W, et al. Current challenges for unseen-epitope TCR interaction prediction and a new perspective derived from image classification [published online ahead of print, 2020 Dec 21]. Brief Bioinform. 2020. doi:10.1093/bib/bbaa318.

15.	Sidhom J-W, Pardoll D, Baras A. AI-MHC: an allele-integrated deep learning framework for improving Class I & Class II HLA-binding predictions. bioRxiv [Preprint]. 2018. bioRxiv 318881. Available from <https://doi.org/10.1101/318881>.

17.	Liberis E, Veličković P, Sormanni P, Vendruscolo M, Liò P. Parapred: antibody paratope prediction using convolutional and recurrent neural networks. Bioinformatics. 2018;34(17):2944-50.

We added the following text to the Introduction on pages 4-5, lines 110-117:

“*Artificial Intelligence (AI) methods are potentially well suited to address these types of problems (8). Thus, machine learning (ML) approaches have already been used to analyze and classify information derived from cells expressing adaptive immune receptors (9). Some of these ML applications include predicting peptide presentation by T cells (10-13), affinity of peptide binding to Major Histocompatibility Complex molecules (14, 15), and binding affinity of neutralizing Abs (16).Additionally, deep learning techniques have also been used in Ab paratope prediction (17).”*

“The epitope binding site on the antigen itself is not used as we typically do not have this information.” → If you don’t have the epitope, you, by definition, also don’t have the paratope. How was the paratope delineated for the Ab sequences studies?

To clarify this issue, we rephrased these sentences and page 5, lines 130-133, now reads:

“*The fingerprints are derived from 3-D models representing the unbound conformation of an Ab. No attempt was made to account for excluded volume effects on the CDR loops due to the presence of antigens*.”

Can you provide an overview table with the number of Abs in Train, Val, and Test datasets? This paper contains a lot of tables. Can you maybe summarize them all together in one figure using boxplots/barcharts. In such a figure, you could then also visualize the research of a given ML experiment, which would make this manuscript much clearer. It's overall very confusing to read.

We agree that the extensive effort presented here may be challenging, yet it does not lend itself to an easy simplification. We have added an overview of the experiments we conducted in Table 1 (page 8, lines 214-216) – similar in spirit to the removed introductory Figure, but containing a more detailed and quantitative summary of the conducted experiments. This covers the type of tests, the number of Abs used, a summary of the training and validation, and the evaluation of the testing set.

Can the authors compare their method to already existing approaches such as: <https://academic.oup.com/bioinformatics/article/34/17/2944/4972995> or with simpler baseline approaches as Logistic regression? Or at least discuss why they did not compare with prior literature?

As we mentioned in the response to Reviewer 2, our method attempts to identify common structural features in clonally distinct Abs that recognize the same epitope through different topologies. It is not our intention to detect residues of the paratope that can be in contact with the antigen, as PARAPRED does. As the authors of PARAPRED mentioned, their methodology is useful to delineate the binding site for applications such as rigid docking.

To clarify the point raised by the Reviewer, we added the following text to the Discussion (page 29, lines 836-849):

“*The ultimate goal is to develop a computational method for enriching pools of known disease-specific antibodies through the detection of clonally distinct Abs with similar functionality. In particular, cases where such common functionality cannot be inferred from sequence based analysis. With this objective in mind, we are exploring the use of DNNs to detect common key features on the Abs. We designed a series of applications where we trained DNNs to predict functional characteristics of the Ab, using two-dimensional images of the Ab binding site. No explicit sequence or residue-connectivity information is used during training of the DNN models. These images or fingerprints correspond to reduced representations of Abs that highlight particular properties of the residues forming the binding site such as the net charge distribution using a simple color scheme. It must be noted that the current method is not intended to address problems such as the prediction of single mutation that disrupt binding or destabilize Ab-antigen complexes.*“

Can you provide sequence similarity quantification of the datasets to be classified to clarify the a priori difficulty of the classification challenge? In other words, can you quantitatively motivate why machine learning/deep learning is necessary for your research question?

“1) Can we differentiate Abs based solely on paratope features? 2) Can we identify sets of Abs produced by B cells that originated from a common ancestor, i.e., the family lineage?”
→ ad 1) It’s unclear what this question means? Differentiate based on what?
→ ad 2) Why not use phylogenetic analysis?

The main goal of this work is to produce a methodology to identify Abs with a similar function, but not clonally related. Specifically, cases where the common functionality cannot be infer from a phylogenetic analysis. We attempt to show that the methodology can detect similarities among Abs that share a given property. The fingerprints highlight residues with particular properties, such as the net charge. In the applications we presented, the DNNs are trained with the fingerprint images only. We do not use Ab sequences or residue connectivity to train the DNN models. The challenge arises from the large number of images require to training the DNNs. We documented, in detail, those numbers in the Tables containing the results of each application. For completeness, we have introduced as Supplemental Materials, Table S2 and Table S6, on pages 5-7 and 10, respectively, describing family lineages of the Abs and the amino acid composition of CDRs. Please, note that the Supplemental Materials is now provided as a separate document as requested by the editorial office.

To address this point, we modify the text on page 6, lines 153-156, as follows:

*“Specifically, we aimed to investigate if the developed approach could address the following of four problems without using explicitly sequence information or residue connectivity: 1) Can we identify Abs based solely on a reduced number of features from paratope? 2) Can we identify the family lineage of Abs using features from the paratopes?”*

Can you discuss to what extent your approach can be applied to datasets where extensive lineage information is not available? Is your approach dependent on prior 3D-information?

The methodology does not require information on the Abs lineages, only the sequence of the Ab light and heavy chain. Rosetta Antibody uses a database of high-resolution structures from PDB to generate homology models.

To clarify this issue, we added the following text to the Discussion on page 31, lines 910-913:

*“We must note that this methodology does not use lineages information on the Abs, and sequence information is only used the produce the structural models of the Abs to determine likely distributions of key residues. The Ab sequences are not used to train the DNN models.”*

Figure 1: this figure is trivial. This is not a review. Please add some information that we don’t know already. You are not the only one thinking of image classification: <https://www.biorxiv.org/content/10.1101/2019.12.18.880146v2>

We have followed the Reviewer’s suggestion and moved Figure 1 to the Supplemental Materials. We also included a reference to the indicated preprint in the Introduction (page 5, line 114):

13.	Moris P, De Pauw J, Postovskaya A, Gielis S, De Neuter N, Bittremieux W, et al. Current challenges for unseen-epitope TCR interaction prediction and a new perspective derived from image classification [published online ahead of print, 2020 Dec 21]. Brief Bioinform. 2020. doi:10.1093/bib/bbaa318.

Minor

The human immune system is capable of producing on the order of 10^20 antibodies (Abs) in response to a viral infection. → citation for 10^20?

We have now provided this information in the manuscript. Briefly, normal levels of IgG in human serum range from 7 to 15 g/L. Given that the human body contains approximately 3 liters of serum, the amount of IgG on an individual with the highest normal IgG level, T(IgG), will be approximately 45 g. On the other hand, given that the molecular weight of an IgG molecule is ~1.5E5 g/mol, the approximate weight for a single molecule, w(IgG), is 2.5E-19 g. Thus, we can get a rough estimate of the number of IgG molecules in the body, N(IgG) as follows: N(IgG) = T(IgG)/ w(IgG) ~ 45g / 2.5E-19g = 1.8E+20. Other immunoglobins are present in blood at much lower concentrations.

Dati F, Schumann G, Thomas L et al. *Consensus of a group of professional societies and diagnostic companies on guidelines for interim reference ranges for 14 proteins in serum based on the standardization against the IFCC/BCR/CAP Reference Material (CRM 470)*. International Federation of Clinical Chemistry, Community Bureau of Reference of the Commission of the European Communities, College of American Pathologists. Eur J Clin Chem Clin Biochem 1996; 34:517–20.
