# Supplementary material for: Using the antibody-antigen binding interface to train image-based deep neural networks for antibody-epitope classification
Source: PLoS Comput Biol. 2021 Mar 29;17(3):e1008864. doi: 10.1371/journal.pcbi.1008864 (PMC8032195; doi:10.1371/journal.pcbi.1008864)
Supplement: Attachment — Submitted filename: Response-to-Editor&ReviewersMAR0521.docx [file pcbi.1008864.s014.docx]

Charlotte M Deane
Associate Editor
PLOS Computational Biology

Nir Ben-Tal
Deputy Editor
PLOS Computational Biology

Thank you very much for submitting your manuscript "Using Paratope Features to Train Deep Neural Networks for Antibody Classification" for consideration at PLOS Computational Biology. As with all papers reviewed by the journal, your manuscript was reviewed by members of the editorial board and by several independent reviewers. The reviewers appreciated the attention to an important topic. Based on the reviews, we are likely to accept this manuscript for publication, providing that you modify the manuscript according to the review recommendations.

We have adopted the recommendations from the review. We modified the original title of the manuscript, and replaced the term “paratope features” within the text to address the Reviewer’s concern. Below, we provide a detailed response to the comments of Reviewer #1. We hope that these modifications renders the manuscript editorially acceptable without further rounds of revisions.

Response to Reviewer Comments

Reviewer #1: I thank the authors for making significant improvements to the design of their investigation, in particular removing any sequence redundancy and adding a crucial OCC experiment where the normal class compromises different-lineage antibodies that engage the same binding site. The results act as a proof-of-concept that their methodology (at least in 4-6% of models) can perform the difficult task of "seeing past" lineage to bin together less related antibodies able to compete for the same epitope.

We agree that this point was missing in the original submission and that we now have better arguments to support the proposed approach. Part of this analysis also highlighted that although the overall net positive predictive value is modest, we correctly identified all decoy as decoys, and among the ~40 Abs targeted for detection we identified 4-6, i.e., ~10%.

While this tool shows promise, its typical performance (and in particular its significantly lower performance than sequence-based methods at binning same-lineage same-epitope binders together) is likely to limit its immediate wider adoption by the community. Nonetheless, this drop in performance is perhaps unsurprising given the need to relax the similarity threshold to capture more distantly-related antibodies that bind the same epitope with different binding modes. This paper represents a thorough and useful initial benchmark of the performance of a 2D image-based DNN for antibody epitope binning.

Again, we appreciate the Reviewer’s acknowledgement of our efforts to establish the proof-of-principle and create a non-trivial benchmark using a new technology. The key is that we can identify distantly-related antibodies – which ultimately could be used for identifying novel therapeutic antibodies.

I would be interested to see just how much the performance improves when trained on binders to more intensely studied epitopes, such as the large number of antibodies now shown to compete for the ACE-2 binding site of the SARS-CoV-2 spike receptor binding domain.

We agree with the Reviewer on this point. We are currently exploring the applicability of our methodology to identifying and classifying anti-SARS-CoV-2 Abs. A recent publication from Ian Wilson’s group at Scripps reported three main binding sites on the Receptor Binding Domain (RBD) of the spike protein of SARS-CoV-2 Abs. Our preliminary inspection of the existing Abs complexes in PDB indicates that, beside electrostatic charge-charge interactions, pi–pi interactions may play a crucial role in Ab recognition of the RBD epitopes. We are exploring how to account for these interactions in our methodology.

Overall, I could see this published in PLoS Computational Biology as a valuable initial yardstick against which to measure future structure-aware antibody function classification algorithms.

We appreciate the Reviewer’s comment, and hope that this work will become an important stepping-stone in the development of algorithms for structure-aware antibody function classification.

As an aside, I agree with reviewer 3 that using the term “paratope features", in the title and throughout the manuscript, is too strong as you are neither predicting nor evaluating the paratope ahead of feature selection. From your graphic, it appears that you are equating the term 'paratope' with the CDRs. In addition, the use of the term “classification” in the title, while accurate, does not communicate the ultimate intended application of the method. Since you settled on an APN residue alphabet, I would suggest the following title: “Using the Spatial Distribution of Complementarity-Determining Region Charges to Train Deep Neural Networks for Antibody Epitope Binning”, or some variation thereof.

It is a point well-taken, we have somewhat loosely used the “spatial distribution of key residues on the Complementarity-Determining Regions and vicinity” to signify “paratope features.” This could be misconstrued, and although the ultimate intended application may still have to be settled on, we believe a change of title could be warranted. We did explore more features that using just the charged alphabet – the method can still be implemented using different alphabets (also see the above comments on possible extension to include pi-pi stacking interactions) – and trying a compromise between being too inclusive (the original title) to something that is so specific to render the title inaccessible to a general audience, we settled on “Using the Antibody-Antigen Binding Interface to Train Image-Based Deep Neural Networks for Antibody-Epitope Classification.”

In addition and for consistency, we changed the term “paratope” in multiple places of the manuscript when necessary. We replaced it with terms such as “antibody-antigen binding interface” or “antibody binding site region.” These changes are highlighted in one of the revised copies of the manuscript that we uploaded.

Reviewer #3: All of my comments have been addressed.
